# Augurin production in the mammalian choroid plexus: Implications for CSF and hydrocephalus

**DOI:** 10.1186/1743-8454-7-S1-S30

**Published:** 2010-12-15

**Authors:** Sonia Podvin, Xitong Dang, Stuart Knowling, Ana Maria Gonzalez, Miles Miller, Edward Stopa, Conrad Johanson, Raul Coimbra, Brian Eliceiri, Andrew Baird

**Affiliations:** 1 Surgery Department, University of California San Diego, San Diego CA 92103, USA; 2 Molecular Neuroscience Group, School of Medicine, University of Birmingham, Edgbaston B15 2TT, UK; 3 Biomedical Neurosurgery Department, Brown University, Providence, RI 02912, USA; 4 Department of Pathology, Brown University, Providence, RI 02903, USA

## Background

Whereas augurin, a protein encoded by the ecrg4 gene, is highly conserved across species, bioinformatic algorithms predict the existence of several other potential hormone-like peptide products transcribed from the same gene. With gene expression highest in the mammalian choroid plexus (CP) compared to all other tissue types however, we deemed it critical to know which peptide(s) is produced by the CP so as to determine its potential release into, and activity in, cerebrospinal fluid (CSF). Our previous data has shown that gene knockdown in developing zebrafish causes severe and dose-dependent hindbrain edema/hydrocephalus. Accordingly, we suggested a novel function for ecrg4 gene products in CP physiology and implicated them as new hormone(s) regulating CSF and CP function. Immunohistochemical staining showed protein in CP epithelium in vitro and in vivo, and ligand-targeting shows internalization into ependymal cells.

## Materials and methods

Ecrg4 gene products were detected by immunoblotting and immunofluorescence with chicken and rabbit polyclonal or mouse monoclonal antibodies that we raised. DNA sequences for fragments (31-148), (31-70) and (71-148) were cloned into pET15b vector, expressed in BL21DE3pLysS and purified. The human gene was cloned into the pLVx-IRES-ZsGreen vector and virus generated using the LentiX kit (Clontech) while siRNA lentivirus was obtained from Santa Cruz Biotechnology. Human primary epithelial CP cells were purchased from ScienCell Research Laboratories.

## Results

Immunoblotting of CP tissue and lentivirus-transduced primary epithelial CP cells reveals a single 14 kDa immunoreactive band that co-migrates with recombinant augurin [ECRG4(31-148)] indicating that the predicted signal peptide for secretion (ECRG4(1-30)) is removed with no additional predicted processing. Immunostaining of both rodent epithelial CP and primary human CP cells shows that immunoreactivity is present and localized at least in part, to the plasma membrane. These data suggest that, while secreted, augurin may remain cell associated and not released. If so, it may act in an autocrine fashion on CP epithelia and ependyma. Thus, we tested for augurin in conditioned media and found very little peptide, even when over-expressed.

## Conclusions

The presence of the 14 kDa band in the CP and its localization at the cell surface indicates that augurin may be a ligand that is either tethered to the cell surface or secreted and bound to an unidentified receptor on the CP in an autocrine/paracrine fashion. With this in hand, we can begin to develop a model to explain the function of CP-derived augurin in ependyma, the CP and in brain fluid balance.

